# Cultivar-specific drought responses in pineapple revealed by concurrent changes in PSII and PSI energy partitioning

**DOI:** 10.3389/fpls.2026.1826518

**Published:** 2026-06-01

**Authors:** Dongsheng An, Chengming Yan, Junjun He, Zhijun Xu, Junbo Su, Baoshan Zhao

**Affiliations:** 1 South Subtropical Crops Research Institute, Chinese Academy of Tropical Agricultural Sciences/Key Laboratory of Tropical Fruit Biology, Ministry of Agriculture and Rural Affairs of China, Zhanjiang, China; 2 Zhanjiang Experimental Station, Chinese Academy of Tropical Agricultural Sciences, Zhanjiang, China; 3 Guangdong Engineering Technology Research Center of Dryland and Water-Saving Agriculture, Zhanjiang, China; 4 Key Laboratory of Tropical Crops Nutrition of Hainan Province, Haikou, China

**Keywords:** CAM plants, chlorophyll fluorescence, drought stress, non-photochemical quenching, photoprotection

## Abstract

Pineapple (*Ananas comosus (L.) Merr.*), a typical Crassulacean acid metabolism (CAM) crop, exhibits remarkable drought tolerance; however, the concurrent responses of photosystem II (PSII) and photosystem I (PSI) under drought stress remain unclear. In this study, three pineapple cultivars (‘MD-2’, ‘Tainong21’, and ‘Paris’) were subjected to progressive drought stress (mild, moderate, and severe) followed by rewatering, PSII energy allocation and PSI limitation responses was evaluated using Dual-PAM chlorophyll fluorescence measurements. Drought stress significantly altered photosynthetic energy partitioning among cultivars, as reflected by consistent changes in ETR(II), NPQ, and Y(NO) across drought stages. Under mild drought, ‘Tainong21’ showed an early reduction in ETR(II) accompanied by relatively elevated NPQ, whereas ‘Paris’ exhibited a comparatively conservative response by reducing photochemical activity under moderate drought. In contrast, ‘MD-2’ maintained relatively higher ETR(II) together with stable NPQ throughout drought progression and showed the strongest recovery after rewatering. Across cultivars, severe drought was associated with decreased NPQ and increased Y(NO). PSI-related parameters showed comparatively smaller variation, with stable Y(ND) but increased Y(NA) under severe drought. Linear mixed-effects model analysis further indicated that the estimated cyclic electron flow (CEF), used as a proxy, exhibited a significant cultivar × drought interaction under high light conditions. Most fluorescence parameters partially recovered after rehydration, suggesting that the observed limitations were largely reversible at the functional level. Overall, these results demonstrate cultivar-specific photosystem response patterns under drought and provide a physiological basis for fluorescence-based phenotyping and drought-tolerance screening in CAM crops.

## 1 Introduction

Pineapple (*Ananas comosus (L.) Merr.*) is an important tropical fruit crop cultivated in more than 80 countries, with China being one of the major producers, producing approximately 2.06 million metric tons in 2022 from 66.7 thousand hectares. The pineapple industry plays an important role in supporting rural economies, with a growing processing sector valued at approximately ¥10.3 billion in 2023, fueled by germplasm innovation and precision fertigation protocols that elevated yield ceilings by 18-22% (Liu et al., 2024).

Crassulacean Acid Metabolism (CAM) exemplifies evolutionary optimization for arid and heat adversity through temporal niche partitioning: nocturnal stomatal conductance achieves CO_2_ fixation via phosphoenolpyruvate carboxylase (PEPC)-mediated malate synthesis, while diurnal decarboxylation liberates CO_2_ for Rubisco-mediated Calvin-Benson cycle assimilation. Genomic interrogation of pineapple (typical CAM-model species) uncovers cis-element-mediated co-regulation of CAM pathway genes and circadian transcriptional oscillators (Ming et al., 2015). This diel stomatal regulation slashes transpirational water loss (Osmond, 1978), resulting in relatively high water-use efficiency (WUE), typically reported to range from 10–40 g CO2 kg^-^¹ H_2_O, compared with approximately 2–5 for C_4_ and 1–3 for C_3_ plants (Escamilla-Treviño, 2012). Exceptional drought tolerance is attributed to succulent leaf tissues for water storage and lignified barriers for preventing water back to the soil, which enable CAM species to survive under prolonged aridity (Carr, 2012).

Sustained water deficit in pineapple triggers foliar premature senescence characterized by a sequential progression of chlorosis, anthocyanin accumulation, and necrosis from basipetal to acropetal (Bartholomew et al., 2003, pp. 69-107). Yield penalties manifest as reductions in individual fruit weight, fruit set and approximately 14-18% declines in total soluble solids (Bartholomew et al., 2003, pp. 167-202; Feng et al., 2017). Despite FAO-prescribed crop coefficients (Kc) for irrigation scheduling (Allen et al., 1998), drought–yield relationships in pineapple remain insufficiently quantified, constrained by circadian-mediated decoupling between vapor pressure deficit (VPD) and Rubisco carboxylation kinetics under variable irrigation regimes (Bartholomew et al., 2003, pp. 167–202). A coupled soil–plant–atmosphere modeling framework has been proposed to quantify the interactions between carbon assimilation and water fluxes in CAM photosynthesis (Bartlett et al., 2014). Field trials revealed that regulating root-zone soil water potential to −35–−55 kPa during seasonal droughts sustained pineapple productivity with concurrent >25% fertilizer reduction via fertigation (An et al., 2022). Further validation through coupled water-fertilizer trials established soil moisture availability as the governing factor with mechanistic evidence confirming its dominance over nutrient delivery efficiency (Ma et al., 2022).

Despite mounting evidence confirming water availability as a crucial regulator of pineapple yield formation and biochemical quality attributes, approximately 80% of pineapple cultivation remains rain-fed (Bartholomew et al., 2003, pp. 136-137), a pattern equally prevalent in China. This underscores the critical need to identify pineapple water status. Root-mediated drought-sensing mechanisms initiate signaling cascades (e.g., ABA, ROS) that modulate the photosynthetic apparatus (Sarwat and Tuteja, 2017), thus non-destructive photosynthetic phenotyping enables rapid screening of drought-tolerant pineapple cultivars with high photosynthetic efficiency as an optimal approach for drought stress diagnosis. The inherent diurnal decoupling of photosynthetic processes in CAM species compromises conventional gas exchange metrics, a mainstay in C_3_/C_4_ crop evaluation frameworks for photochemical efficiency and drought resilience (Feng et al., 2024; Abu-Ria et al., 2024), while elevating chlorophyll fluorescence as the preferential phenotyping modality in pineapple research.

Recent studies have demonstrated that chlorophyll fluorescence provides a sensitive, non-destructive indicator for diagnosing drought-induced perturbations in the photosynthetic apparatus across diverse plant species (Chang et al., 2025). These studies emphasize that fluorescence-derived parameters effectively capture changes in photochemical efficiency and energy dissipation that are not readily resolved by gas exchange measurements alone. Maximum quantum efficiency (*F*_v_/*F*_m_), non-photochemical quenching (*NPQ* or *q*_N_), the quantum yield of PSII (*Φ*_PSII_ or *Y*_II_), fast chlorophyll fluorescence induction curves (OJIP) and other specific energy fluxes (*ABS*/*RC*, *Tr0*/*RC*, *Di0*/*RC*, *Et0*/*RC*, *Re0*/*RC*, etc.) have emerged as pivotal indices for pineapple and other CAM plants in the study of photosynthetic efficiency (Ritchie and Bunthawin, 2010), photo-inhibition (Kornas et al., 2009), drought stress (Herppich et al., 1997; Fondom et al., 2014; Ceusters et al., 2019), saline irrigation (Brito et al., 2020), etc.

Moreover, recent analysis of photosystem vulnerabilities under abiotic stresses highlights that PSII and PSI exhibit distinct sensitivities and protective strategies, and that their interactions play an important role in maintaining photochemical stability under drought conditions (Gao et al., 2025). These insights suggest that PSII-centered assessments alone may be insufficient to fully characterize stress responses of the photosynthetic machinery. However, existing studies in pineapple and other CAM crops have predominantly focused on PSII-related responses, while the behavior of PSI under drought stress remains largely unexplored.

Although PSII-related fluorescence parameters have been extensively applied and have greatly advanced our understanding of stress responses in CAM plants, they primarily reflect PSII photochemistry and energy dissipation. In contrast, PSI-related parameters, such as Y(ND) and Y(NA), which provide insight into donor- and acceptor-side limitations of PSI, have been far less investigated in pineapple and other CAM species. This imbalance limits a comprehensive understanding of photosystem responses under drought stress.

In this study, we conducted a systematic assessment of PSII and PSI energy metabolism in plants of three pineapple cultivars subjected to progressive drought stress (mild, moderate, and severe) followed by rehydration. By integrating Dual-PAM chlorophyll fluorescence measurements, we comparatively analyzed drought-induced photochemical limitations, energy dissipation strategies, and post-rehydration recovery dynamics across cultivars. This approach enabled the identification of cultivar-specific photoprotective strategies and differential recovery capacities, thereby elucidating the concurrent changes of PSII and PSI during drought stress and subsequent rewatering in pineapple. Collectively, this work provides an energy-partitioning-based perspective for characterizing drought adaptation in pineapple and offers a physiological foundation for developing non-destructive, fluorescence-based indicators for water status diagnosis and drought-tolerance screening in CAM crops.

## 2 Materials and methods

### 2.1 Plant materials and experimental design

A controlled pot experiment was conducted in a glass chamber at the observation and experimental station for the tropical agricultural environment and efficient water use of crops (21.16°N, 110.3°E) from Sep. 2023 to Jan. 2024. Three commercial pineapple cultivars (Ananas comosus var. ‘MD-2’, ‘Tainong21’, and ‘Paris’) were selected based on their economic significance and adaptive characteristics in Zhanjiang, the main cultivation area of pineapple in China. Vegetatively propagated pineapple (Ananas comosus) side shoots (about 20cm length) of three cultivars - ‘MD-2’, ‘Tainong21’, and ‘Paris’ - were established in standardized cylindrical containers (upper diameter 30 cm, base diameter 23 cm, height 28 cm) containing a growth substrate of lateritic soil: organic fertilizer (9:1, v/v). Electrical-thermal fans were activated when indoor temperature fell below 15°C while evaporative cooling with wet curtain and fans system were initiated when it exceeded 28°C.

The three cultivars (‘MD-2’, ‘Tainong21’, and ‘Paris’) were selected based on preliminary observations in a germplasm nursery under natural seasonal drought conditions, where consistent phenotypic differences among cultivars were observed. ‘Tainong21’ exhibited pronounced leaf chlorosis under drought, indicating higher sensitivity to water deficit, whereas ‘MD-2’ maintained greener leaves and showed rapid recovery following rainfall, suggesting stronger drought tolerance. In contrast, ‘Paris’ displayed reduced growth and partial leaf reddening, indicative of a more conservative or drought-avoidance response. These contrasting phenotypic patterns provided a practical basis for selecting representative cultivars with distinct drought-response characteristics for controlled experiments.

A total of 30 potted plants were initially established for each cultivar to account for variability during the slow establishment phase after transplanting, which is common in pineapple plants. Because substantial heterogeneity in growth rate occurs during this stage, plants exhibiting median growth performance were selected to minimize background variation before drought treatments were imposed. From the initial population, ten plants with uniform growth status were retained for subsequent experimental use. Among them, three independent plants were designated as biological replicates (n = 3), and each plant was maintained as a fixed experimental unit throughout the entire drought–rewatering cycle. The same individual plants were repeatedly measured across all drought stages, allowing direct tracking of physiological responses within each biological replicate throughout the experiment. One additional pot per cultivar was equipped with a soil moisture waveguide sensor for measurement of soil water content.

**Figure 1:**
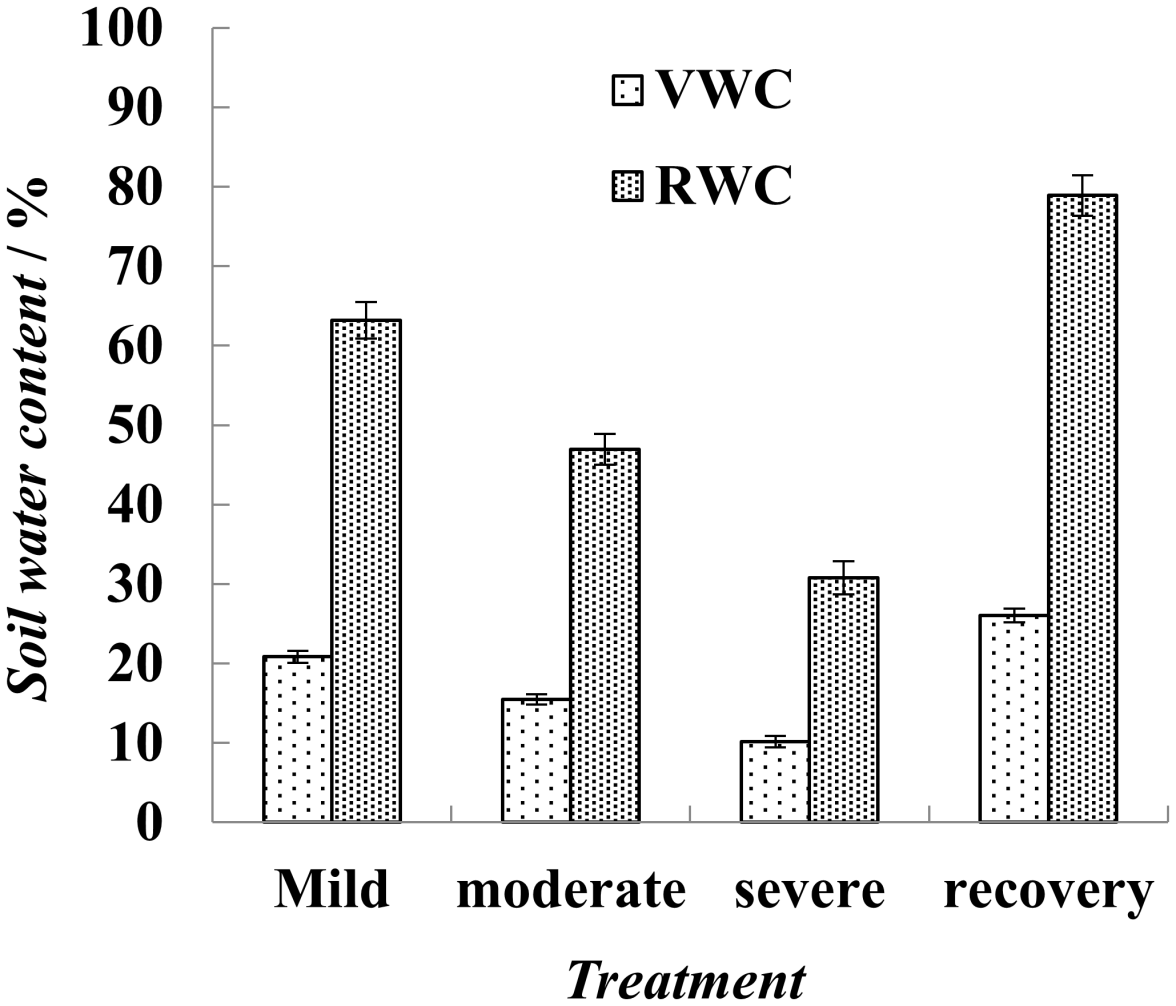
Soil water status under different drought treatments. Soil water content expressed as volumetric water content (VWC) and relative water content (RWC) under four treatments: mild drought, moderate drought, severe drought, and recovery after rewatering.

All plant materials were planted on September 30th, 2023 and the treatments were initiated on November 22nd, 2023. Ten uniform plants from each cultivar were selected and subjected to progressive soil drying by withholding irrigation for 24 days, after which plants were rewatered and plants were maintained under well-watered conditions for an additional 21 days to allow physiological recovery. Measurements were performed at 6, 13, and 24 days after irrigation was withheld, when the relative soil water content (*RSWC*) declined to 63.2 ± 2.31%, 47 ± 1.95% and 30.8 ± 2.07%, corresponding to mild, moderate, and severe drought stress, respectively. After rewatering to 75–80% RSWC, plants were maintained for 21 days before recovery measurements were taken (**Figure 1**). Data were collected on November 27, December 4, and December 15, 2023, and January 5, 2024.

### 2.2 Environmental monitoring

**Figure 2:**
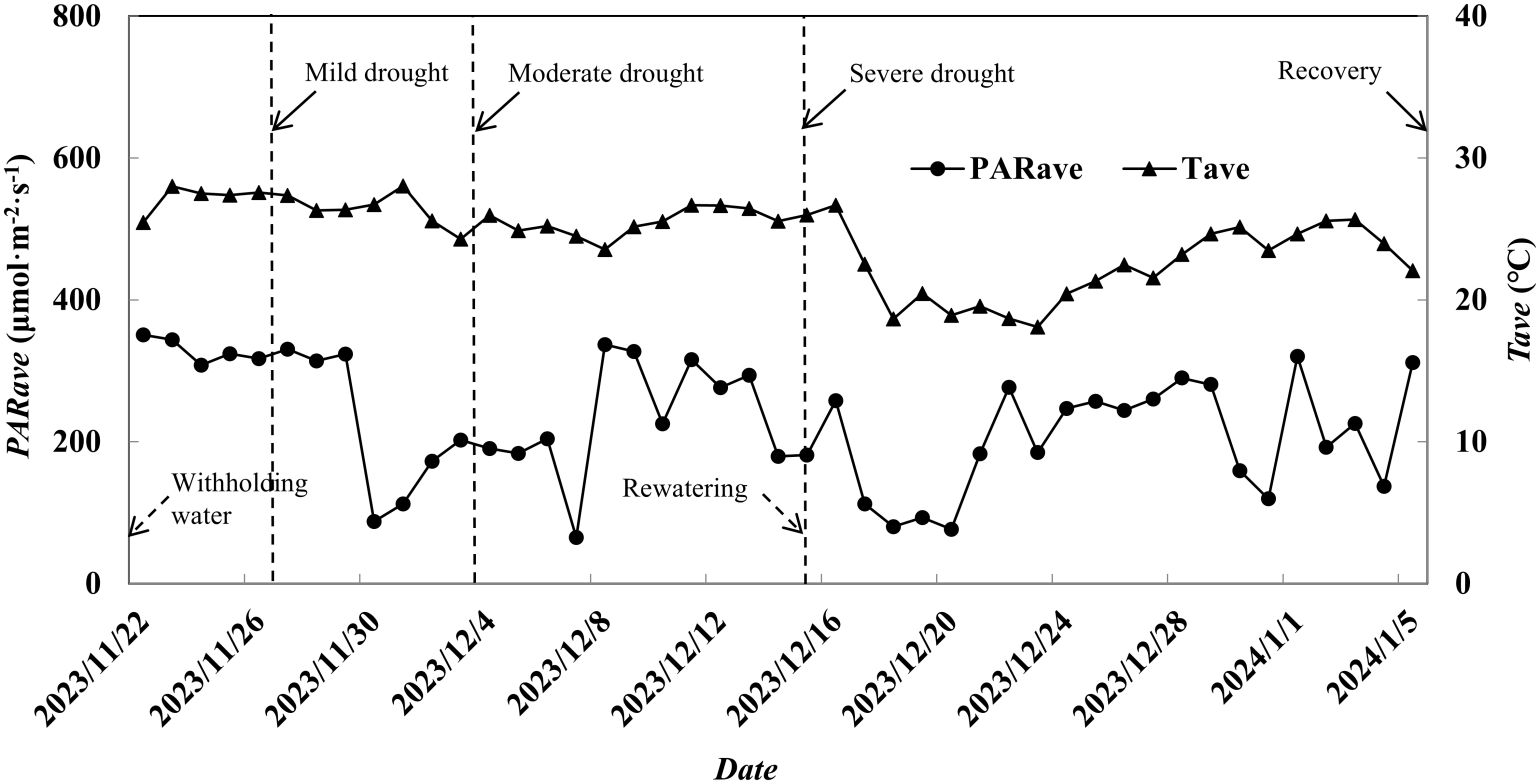
Environmental light and temperature conditions during the experiment. Daily mean photosynthetically active radiation (*PAR*_ave_) and air temperature (*T*_ave_) recorded in the greenhouse throughout the drought and recovery treatments. Vertical dashed lines separate the mild, moderate, and severe drought phases and the recovery stage. Solid arrows denote the sampling dates for chlorophyll fluorescence measurements, and dashed arrows indicate the timing of irrigation treatments (withholding water and rewatering).

Environmental variables were continuously monitored using a Campbell Scientific data acquisition system (Campbell Scientific Inc., Logan, UT, USA). Air temperature and photosynthetically active radiation (PAR) were recorded automatically, and daily mean values were calculated for subsequent analysis (**Figure 2**).

Soil volumetric water content (VWC) was measured using a MiniTrase soil moisture sensor (IMKO GmbH, Ettlingen, Germany). Soil moisture was monitored throughout the experiment, with measurement frequency adjusted according to drying dynamics. Measurements were conducted more frequently during periods of rapid soil drying and near target drought thresholds, and less frequently under stable conditions.

Drought stages were defined based on soil relative water content (RSWC), and measurements were taken when RSWC approached the predefined thresholds corresponding to mild, moderate, and severe drought. To ensure consistency, measurements were performed within a similar time window during the day.

### 2.3 Chlorophyll fluorescence measurements and parameter calculations

Simultaneous measurements of PSII and PSI parameters were conducted using a Dual-PAM-100 system (Heinz Walz GmbH, Germany) in Dual PAM-F&P700 mode under the specifically adjusted environmental conditions for each treatment. Repeated fluorescence measurements were conducted on the same tagged leaves of each biological replicate throughout the experimental period.

Prior to dark-adapted parameters measurement, the corresponding potted plants were kept in complete darkness for 30 min to ensure full oxidation of the photosynthetic reaction centers. Then stepwise increasing actinic irradiance (0, 33, 91, 169, 269, 418, 608, 756, 920, 1175, 1452 and 1806 μmol·m^-^²·s^-^¹) was applied with 90 s acclimation at each light level using three independent biological replicates per cultivar throughout the entire treatment period. Measurements were performed on the distal portion (40-50% from the leaf tip) of the youngest fully expanded leaves (D-position leaves).

Quantified parameters included electron transport rate of PSII (*ETR(II)*) and PSI (*ETR(I)*), non-photochemical quenching in PSII (*NPQ*), non-regulated energy dissipation in PSII (*Y(NO)*), quantum yield of non-photochemical energy dissipation due to donor-side limitation (*Y(ND)*) and acceptor side limitation (*Y(NA)*), Cyclic electron flow (CEF) was estimated as: *CEF* = *ETR(I)* - *ETR(II)*, and used as an indicator of relative changes rather than a direct measurement (Genty et al., 1996; Kramer et al., 2004; Klughammer and Schreiber, 2008; Huang et al., 2015).

### 2.4 Statistical analysis

Data analyses were performed using Microsoft Excel 2019 (Microsoft Corp., Redmond, WA, USA) and Python 3 (Python Software Foundation, Wilmington, DE, USA). Each plant (one plant per pot) was treated as an independent biological replicate (n = 3 per cultivar). Because the same plants were measured repeatedly across drought stages, the dataset contains a repeated-measures structure. Values shown in the figures represent means of three biological replicates, and error bars indicate standard deviations (SD).

Light-response curves were used primarily to visualize overall treatment trends in photosystem activity across actinic irradiance levels. To facilitate statistical comparison while avoiding overinterpretation of repeated measurements across sequential light steps, three representative irradiance levels were selected for significance testing: low light (169 μmol·m^-^²·s^-^¹), medium light (920 μmol·m^-^²·s^-^¹), and high light (1806 μmol·m^-^²·s^-^¹). These irradiance levels correspond to distinct phases of the light-response curve and allow simplified comparison among cultivars and drought treatments.

At these representative irradiance levels, differences among cultivars were evaluated using one-way analysis of variance (ANOVA), followed by Tukey’s HSD test at P < 0.05. These comparisons were conducted within each drought stage and light condition. Heatmaps were generated to visualize relative variations in chlorophyll fluorescence parameters [*ETR(II)*, *NPQ*, *Y(NO)*, *ETR(I)*, *Y(ND)*, and *Y(NA)*] across cultivars, drought treatments, and light intensities.

To account for repeated measurements taken on the same plants across different treatment time points (i.e., drought progression and rewatering stages) and the associated temporal correlation, linear mixed-effects models (LMMs) were additionally applied. Cultivar and drought stage were treated as fixed effects, while plant identity was included as a random effect. LMM analyses were conducted separately for each light intensity to reduce model complexity. Due to the limited number of biological replicates and the variability structure of certain parameters, simplified models were adopted where necessary to ensure model convergence.

Z-score normalization and principal component analysis (PCA) were applied to explore integrated variation patterns among fluorescence parameters across cultivars and drought treatments. These multivariate analyses were used primarily for visualization and exploratory interpretation of overall response patterns rather than for formal hypothesis testing.

## 3 Results

### 3.1 Alterations in PSII photochemical activity of three pineapple cultivars under varying drought intensities

**Figure 3:**
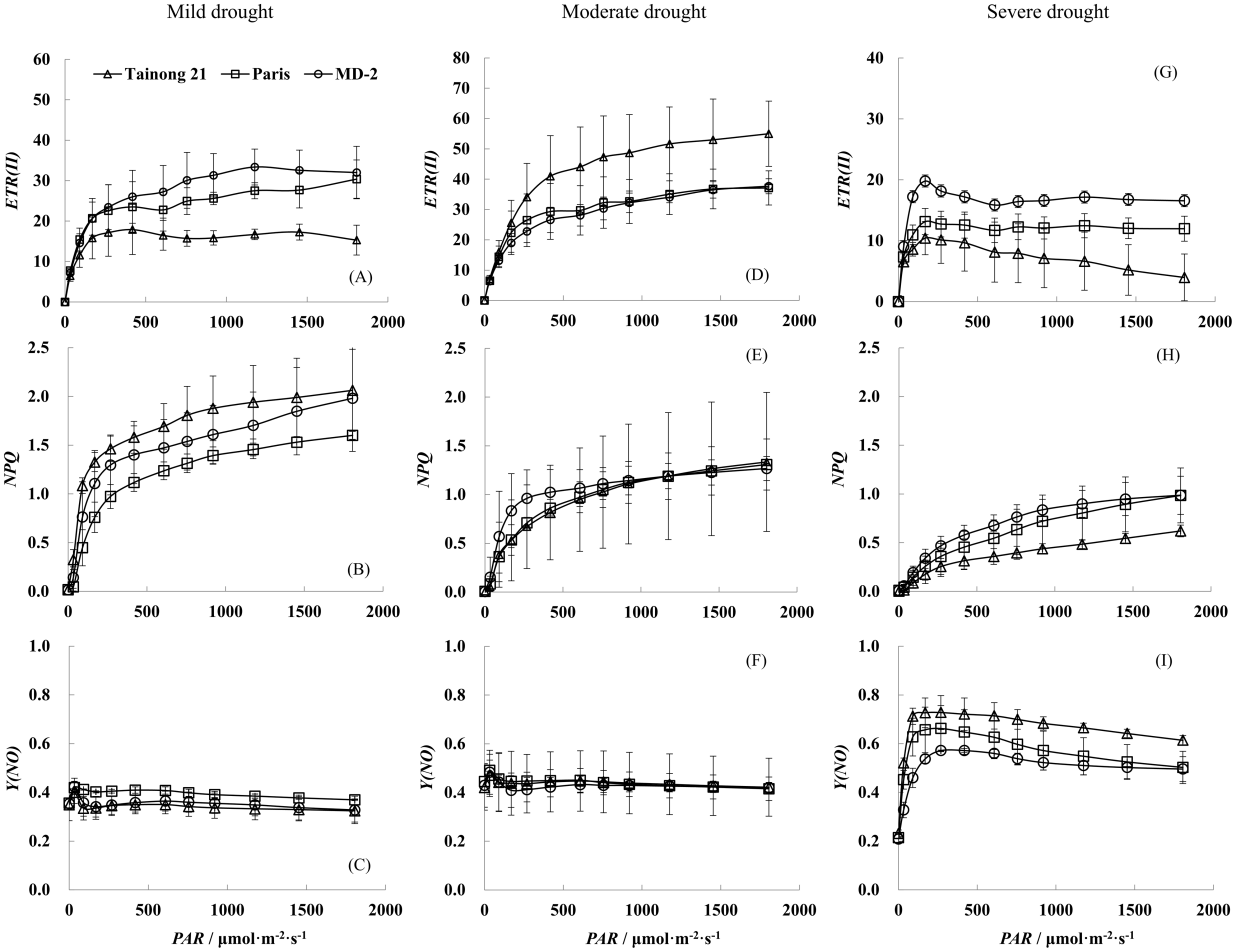
Responses of PSII-related parameters to increasing light intensity under different drought levels. Changes in PSII electron transport rate [*ETR(II)*], non-photochemical quenching (*NPQ*), and non-regulated energy dissipation [*Y(NO)*] in three pineapple cultivars (‘Tainong21’, ‘Paris’, and ‘MD-2’) under mild **(A–C)** moderate **(D–F)** and severe drought **(G–I)**. Measurements were conducted under increasing photosynthetically active radiation (PAR).

Under mild drought, *ETR(II)* increased with increasing irradiance in all three cultivars and gradually approached saturation (**Figure 3A**). Statistical comparisons at representative irradiance levels showed that cultivar differences were relatively small under low light (169 μmol·m^-^²·s^-^¹), whereas under medium and high light (920 and 1806 μmol·m^-^²·s^-^¹), ‘MD-2’ and ‘Paris’ generally maintained higher *ETR(II)* than ‘Tainong21’. *NPQ* also increased with irradiance in all cultivars (**Figure 3B**). Under mild drought, NPQ showed only limited variation among cultivars, with relatively small differences at low light and no significant differences at medium or high light. *Y(NO)* remained at comparable levels among cultivars, although ‘Paris’ tended to show slightly higher values than ‘MD-2’ and ‘Tainong21’ (**Figure 3C**).

Under moderate drought, *ETR(II)* still increased with irradiance in all cultivars, although cultivar-specific differences became less consistent across the light-response curve (**Figure 3D**). ‘Tainong21’ tended to show relatively higher ETR(II) under some light conditions; however, differences among cultivars at the representative irradiance levels were generally not statistically significant. *NPQ* and *Y(NO)* also showed similar patterns among cultivars under moderate drought, with no significant differences detected at the representative irradiance levels (**Figures 3E, F**). Compared with mild drought, *NPQ* generally decreased, whereas *Y(NO)* showed a slight increase.

Under severe drought, *ETR(II)* initially increased and then declined at high irradiance in all cultivars, indicating stronger photochemical limitation under severe water deficit (**Figure 3G**). At the representative irradiance levels, cultivar differences became more evident. Under medium and high light (920 and 1806 μmol·m^-^²·s^-^¹), ‘MD-2’ generally maintained higher *ETR(II)* than the other two cultivars, whereas ‘Tainong21’ showed the lowest values. *NPQ* further declined under severe drought, but ‘MD-2’ and ‘Paris’ tended to maintain relatively higher *NPQ* than ‘Tainong21’, particularly under medium and high light (**Figure 3H**). In contrast, *Y(NO)* showed an opposite tendency, with ‘Tainong21’ displaying relatively higher values than the other cultivars under medium and high light (**Figure 3I**). These results suggest increased non-regulated energy dissipation under severe drought, particularly in ‘Tainong21’, indicating a potential reduction in photoprotective efficiency.

### 3.2 PSI functionality and cultivar-specific responses under differential drought regimes

**Figure 4:**
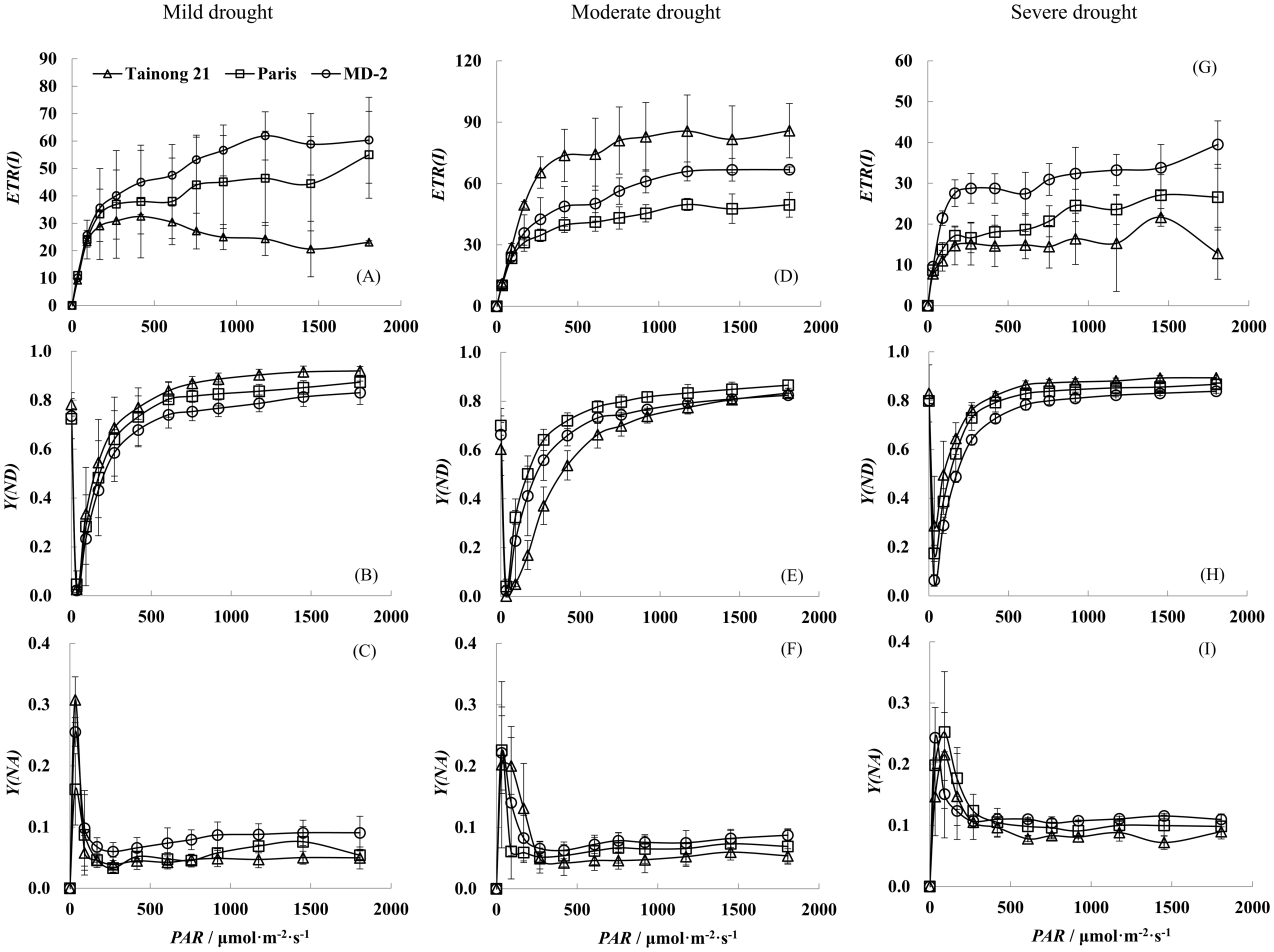
Responses of PSI parameters to increasing light intensity under different drought levels. Light-response curves of PSI electron transport rate [*ETR(I)*], donor-side limitation [*Y(ND)*], and acceptor-side limitation [*Y(NA)*] in three pineapple cultivars (‘Tainong21’, ‘Paris’, and ‘MD-2’) under mild **(A–C)** moderate **(D–F)** and severe drought **(G–I)**.

*ETR(I)* exhibited trends generally similar to those observed for *ETR(II)* (**Figures 4A, D, G**). Across drought treatments, the overall ranking was ‘MD-2’ > ‘Paris’, while ‘Tainong21’ was lower than the other two cultivars under mild drought (**Figure 4A**). Under moderate drought, ‘Tainong21’ exhibited significantly higher *ETR(I)* than ‘Paris’ across all light intensities, and was significantly higher than ‘MD-2’ only under low light (**Figure 4D**). Compared with *ETR(II)*, *ETR(I)* displayed greater fluctuations with increasing *PAR*.

*Y(ND)* levels varied among cultivars depending on light intensity under moderate and severe drought (**Figures 4B, E, H**), and were generally lower than those under mild drought. ‘Tainong21’ exhibited the highest *Y(ND)* under mild drought (**Figure 4B**) but tended to be lower under moderate drought, particularly at low and medium light intensities (**Figure 4E**). Across most drought intensities and light conditions, *Y(ND)* in ‘Paris’ was generally higher than in ‘MD-2’.

*Y(NA)* levels were generally similar among cultivars under mild and moderate drought (**Figures 4C, F**), though some significant differences were observed under specific light conditions, and increased under severe drought (**Figure 4I**). When *PAR* exceeded 500 μmol·m^-^²·s^-^¹, *Y(NA)* followed the order ‘MD-2’ > ‘Paris’ > ‘Tainong21’, with significant differences detected under mild and moderate drought, while differences were not statistically significant under severe drought and after rewatering.

### 3.3 Recovery characteristics of the photosynthetic apparatus after rewatering among cultivars

**Figure 5:**
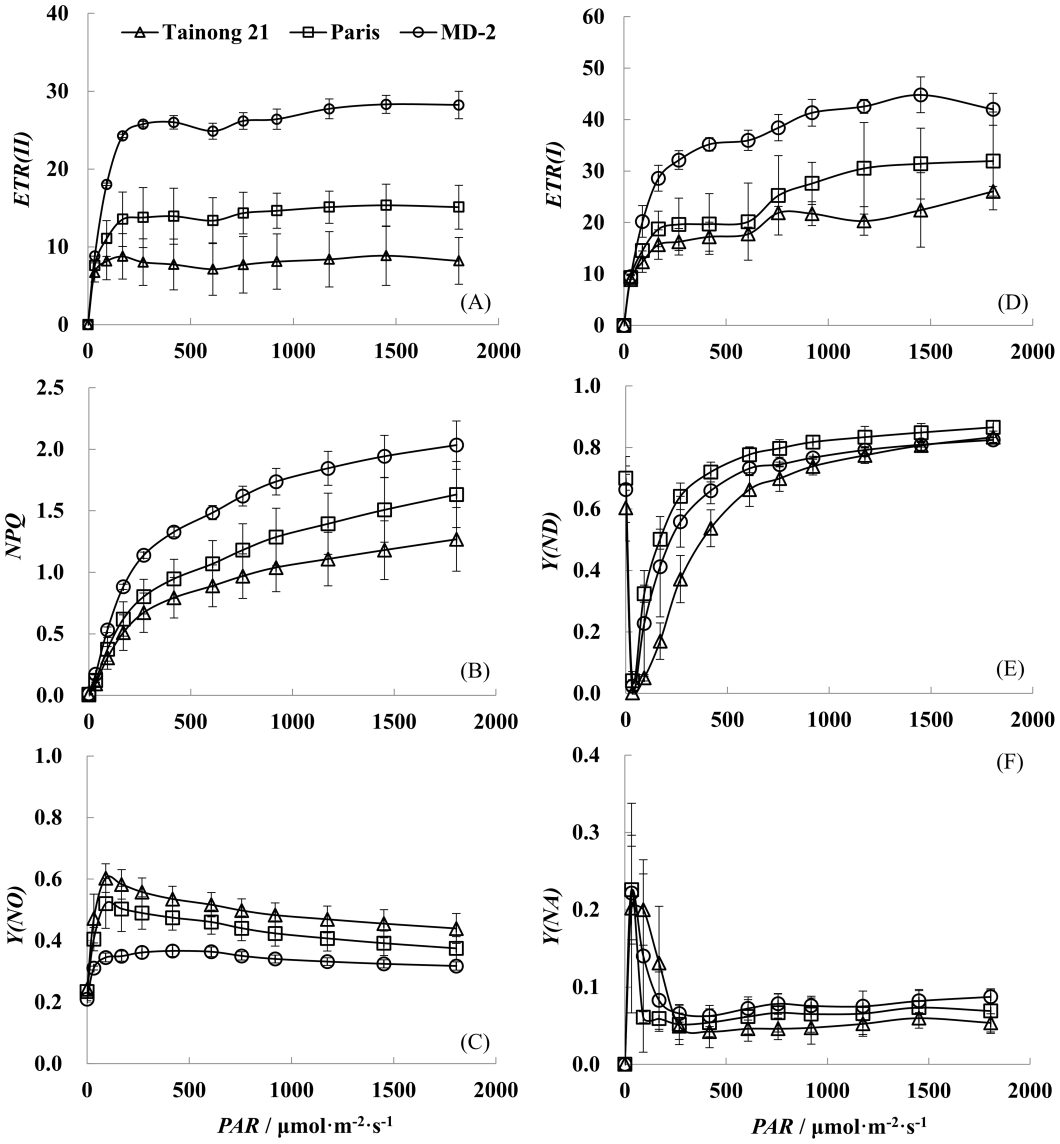
Recovery of PSII and PSI parameters after rewatering. Light-response curves of PSII **(A–C)** and PSI **(D–F)** parameters in three pineapple cultivars (‘Tainong21’, ‘Paris’, and ‘MD-2’) following rewatering.

Following 21 days of rewatering, both *ETR(II)* and *ETR(I)* increased in all cultivars (**Figures 5A, D**). With increasing *PAR*, *ETR(I)* increased gradually, whereas *ETR(II)* rose rapidly and reached saturation. Both parameters ranked as ‘MD-2’ > ‘Paris’ > ‘Tainong21’, with ‘MD-2’ significantly higher than both ‘Paris’ and ‘Tainong21’. When PAR exceeded 1000 μmol·m^-^²·s^-^¹, *ETR(II)* in ‘Paris’ was significantly higher than in ‘Tainong21’ (**Figure 5A**), while *ETR(I)* in ‘Paris’ remained significantly higher than in ‘Tainong21’ as well (**Figure 5D**).

*NPQ* increased substantially upon rewatering (**Figure 5B**), with recovery magnitude following the order ‘MD-2’ > ‘Paris’ > ‘Tainong21’; however, no significant differences were detected among cultivars. In contrast, *Y(NO)* significantly decreased upon rewatering (**Figure 5C**), with the magnitude of decline ranking ‘MD-2’ > ‘Paris’ > ‘Tainong21’. Significant differences were observed among cultivars, with ‘MD-2’ exhibiting significantly lower *Y(NO)* than both ‘Paris’ and ‘Tainong21’ across all light intensities. However, neither *NPQ* nor *Y(NO)* recovered to the levels observed under mild drought.

Compared to pre-rewatering conditions, *Y(ND)* remained largely unchanged in ‘MD-2’ and ‘Paris’, whereas it significantly decreased in ‘Tainong21’ (**Figure 5E**). *Y(NA)* declined in all cultivars post-rewatering (**Figure 5F**), although differences were not statistically significant.

### 3.4 Integrated analysis of chlorophyll fluorescence parameters

**Figure 6:**
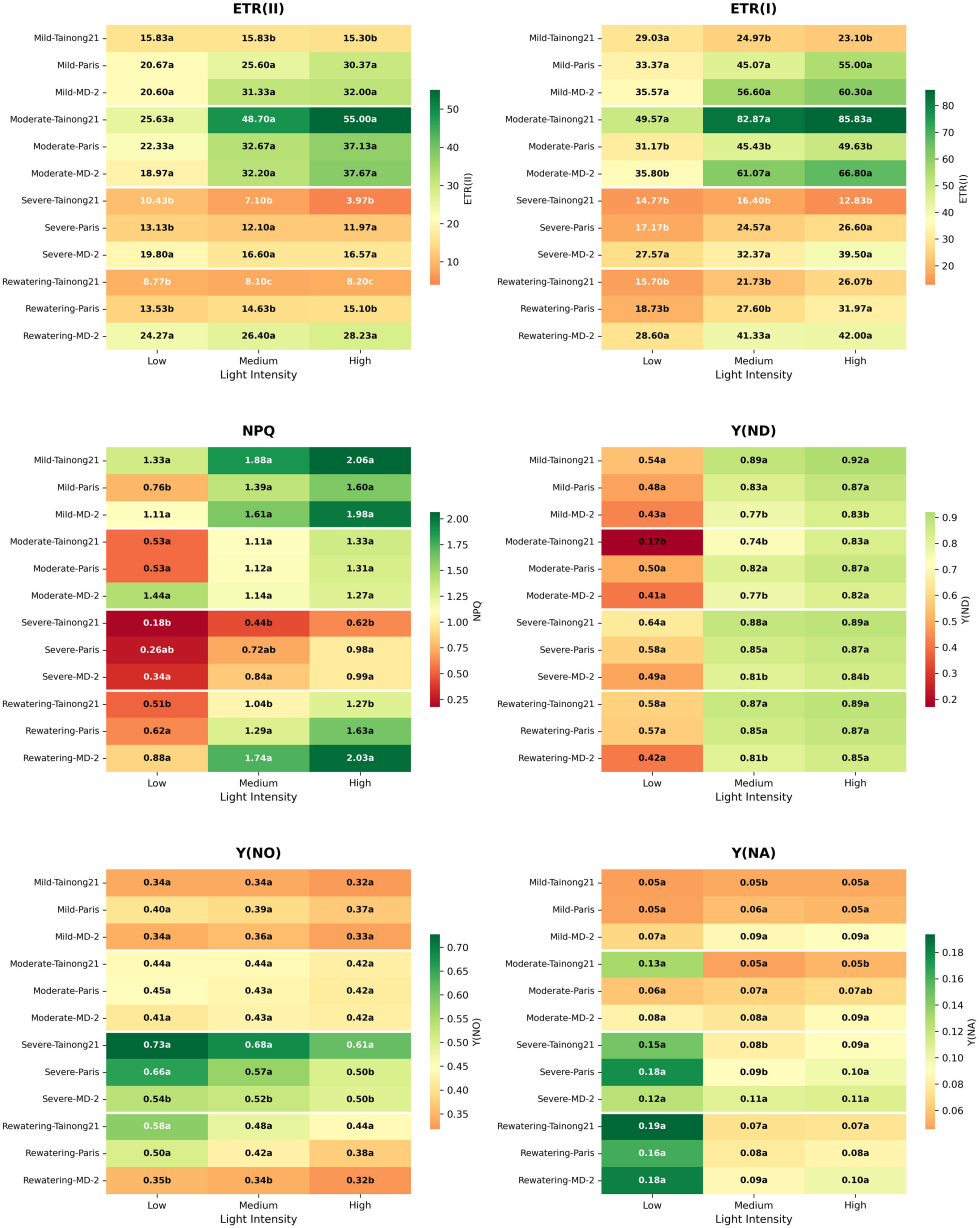
Heatmap of chlorophyll fluorescence parameters in three pineapple cultivars under different drought treatments and light intensities. The heatmap displays mean values of six key chlorophyll fluorescence parameters [ETR(II), NPQ, Y(NO), ETR(I), Y(ND), Y(NA)] in ‘Tainong21’, ‘Paris’, and ‘MD-2’ under mild, moderate, and severe drought, and after rewatering, measured at low, medium, and high light intensities. Each cell represents the mean of three biological replicates, with values color-coded from red (low) to green (high) on a per-parameter scale. Lowercase letters (a–c) indicate significant differences among cultivars within the same drought treatment and light intensity (Tukey’s HSD test, α = 0.05); cultivars sharing the same letter are not significantly different.

To summarize parameter-level differences across cultivars, drought treatments, and light intensities, a heatmap was constructed based on the mean values of six chlorophyll fluorescence parameters [*ETR(II)*, *NPQ*, *Y(NO)*, *ETR(I)*, *Y(ND)*, *Y(NA)*] (**Figure 6**). Each cell represents the mean of three biological replicates, and lowercase letters indicate significant differences among cultivars within the same drought treatment and light intensity (Tukey’s HSD test, α = 0.05).

The heatmap provided an overview of parameter-level variation along the drought gradient. Under mild drought, the three cultivars generally exhibited similar fluorescence characteristics across light intensities. As drought severity increased, cultivar-related differences in several parameters became more apparent. Relatively higher values of *ETR(II)* and *ETR(I)* were often observed in ‘MD-2’, whereas elevated *Y(NO)* values tended to occur more frequently in ‘Tainong21’ under severe drought. In most cases, ‘Paris’ displayed intermediate parameter values between the other two cultivars.

After rewatering, most fluorescence parameters showed partial recovery across cultivars, although some differences among cultivars remained detectable. Overall, the heatmap visualization suggests that increasing drought intensity influenced the distribution patterns of fluorescence parameters across cultivars.

### 3.5 Linear mixed-effects model analysis across light intensities

To address the repeated-measures structure of the dataset, linear mixed-effects models (LMMs) were applied with plant identity included as a random intercept, to account for repeated measurements taken on the same plants across different treatment time points (i.e., drought progression and rewatering stages) and the associated temporal correlation, thereby providing a statistically appropriate framework for analyzing longitudinal responses across drought progression.

The results showed that drought stage significantly affected key parameters such as *ETR(II)*, while cultivar effects were observed for selected parameters depending on light conditions (**Table 1**). Across light levels, drought stage showed consistent effects on *ETR(II)*, particularly under medium and high light conditions, and cultivar effects were also detected, with ‘Tainong21’ differing from the other cultivars. Under medium light, both cultivar and drought affected *Y(NO)*, indicating that non-regulated energy dissipation increased with drought severity and varied among cultivars.

**Table 1:** Summary of linear mixed-effects model results across light intensities.

Light condition	Response	Cultivar	Drought	C × D
Low	ETR(II)	**	ns	—
	NPQ	—	*** (S)	—
	Y(ND)	—	ns	—
Medium	ETR(II)	*	*** (M, S, R)	—
	Y(NO)	**	*** (M, S)	—
	Y(NA)	**	ns	ns
High	ETR(II)	*	*** (M, S, R)	—
	Y(NA)	**	ns	ns
	CEF	***	ns	** (M, R)

Significance codes: ***p < 0.001, **p < 0.01, *p < 0.05, ns = not significant, — = term not included in the converged model. Letters in parentheses indicate which specific drought stages were significant: M, Moderate, S, Severe, R, Rewatering. C × D, Cultivar × Drought interaction. All models include Plant ID as a random intercept. Only converged models are shown.

For PSI-related parameters, cultivar effects were observed for *Y(NA)* under medium and high light, though drought effects were generally not significant. Notably, a significant cultivar × drought interaction was detected for estimated *CEF* under high light conditions, indicating that cultivar-specific differences in this parameter became more apparent under combined high light and drought stress.

### 3.6 Integrated Z-score analysis of photosystem responses under drought

**Figure 7:**
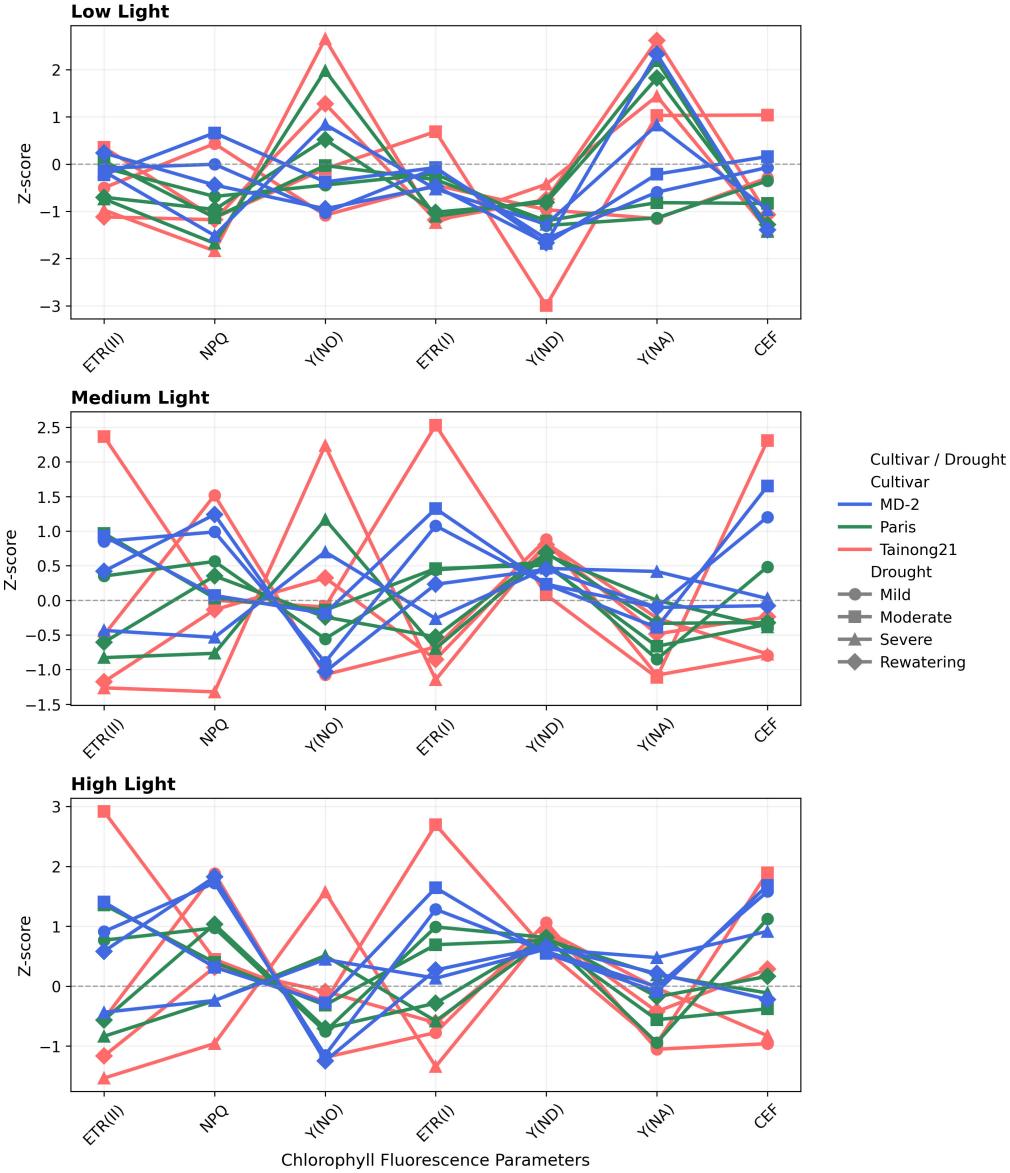
Physiological fingerprint of photosystem parameters in three pineapple cultivars under drought and rewatering. Z-score normalized values of seven chlorophyll fluorescence parameters [ETR(II), NPQ, Y(NO), ETR(I), Y(ND), Y(NA), CEF] in ‘Tainong21’, ‘Paris’, and ‘MD-2’ under mild, moderate, and severe drought, and after rewatering, where estimated CEF was further introduced to evaluate the concurrent changes between PSII and PSI throughout the entire treatment period. Z-score normalization was applied across all samples to enable comparison of parameters with different measurement scales. Positive values indicate above-average expression, negative values indicate below-average expression relative to the overall mean.

To visualize the simultaneous variation of multiple chlorophyll fluorescence parameters across cultivars and drought treatments, Z-score normalization was applied to standardize parameters with different measurement scales (**Figure 7**). This approach allows comparison of relative deviations from the overall mean and provides an integrated visualization of photosystem responses.

Across light environments, the Z-score profiles indicated differences in the relative distribution of fluorescence parameters among cultivars. Parameters associated with photochemical activity, including *ETR(II)* and *ETR(I)*, generally showed higher relative values in ‘MD-2’, whereas non-regulated energy dissipation [*Y(NO)*] tended to be higher in ‘Tainong21’. In most cases, ‘Paris’ exhibited intermediate standardized values between the other two cultivars.

These patterns indicate differences in the distribution of photosystem-related parameters among cultivars. ‘MD-2’ tended to maintain relatively balanced profiles characterized by comparatively higher electron transport together with moderate *NPQ* and lower *Y(NO)*. In contrast, ‘Tainong21’ showed larger fluctuations across several parameters, including elevated *Y(NO)* and variable PSI limitation indices. ‘Paris’ displayed intermediate response patterns, suggesting a comparatively conservative adjustment.

Light intensity also influenced the distribution patterns of standardized parameters. Differences among cultivars were generally more evident under medium and high light conditions, whereas profiles under low light were relatively similar. These observations suggest that cultivar-specific regulation of photosystem activity may become more pronounced when both drought stress and light intensity increase.

### 3.7 Principal component analysis of chlorophyll fluorescence parameters

**Figure 8:**
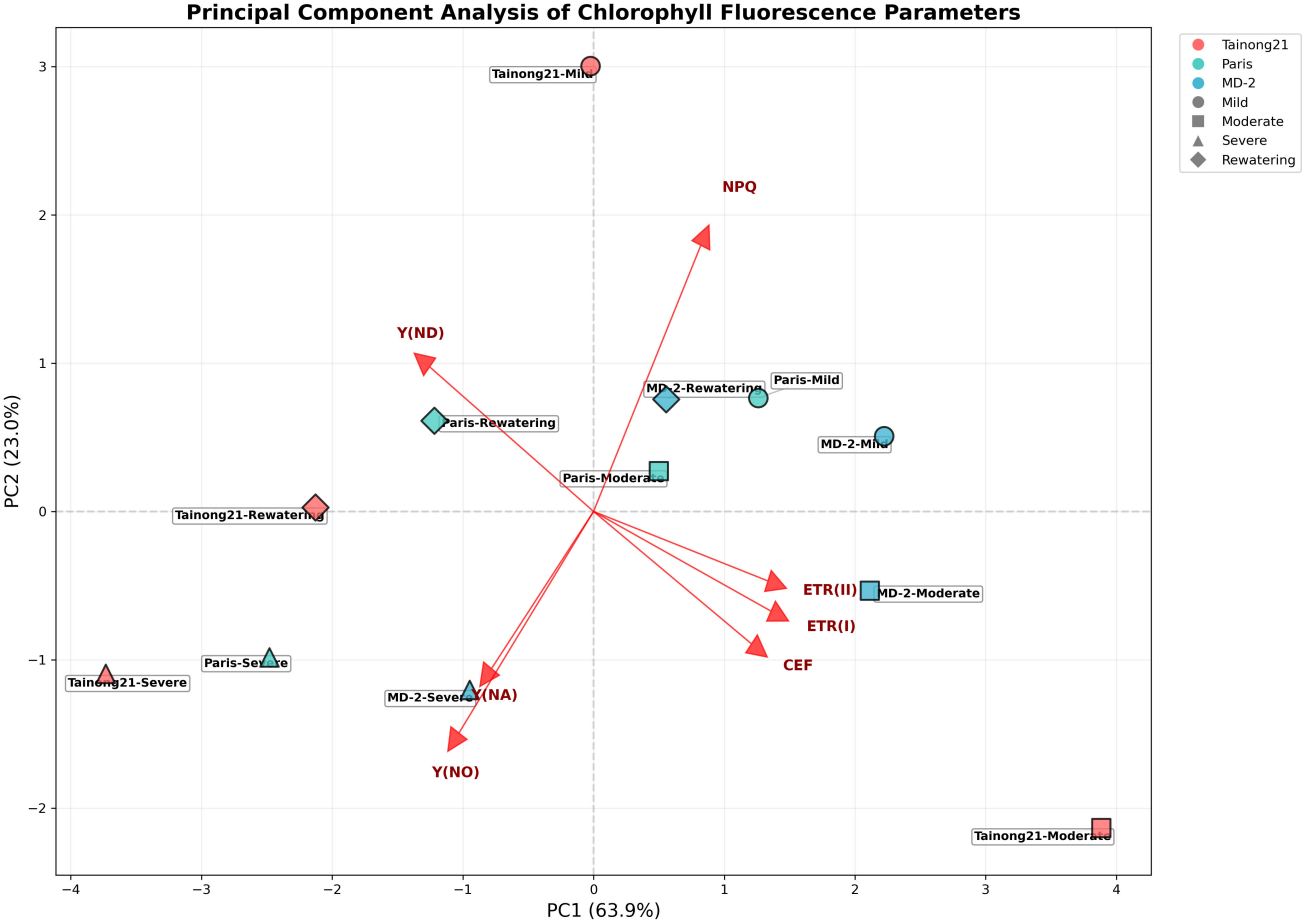
Principal component analysis (PCA) of chlorophyll fluorescence parameters. PCA score plot based on seven chlorophyll fluorescence parameters [ETR(II), NPQ, Y(NO), ETR(I), Y(ND), Y(NA), CEF] measured in three pineapple cultivars (‘Tainong21’, ‘Paris’, ‘MD-2’) under mild, moderate, and severe drought, and after rewatering. The first two principal components (PC1 and PC2) explained 86.9% of the total variance. Arrows indicate the loading vectors of individual parameters. Each point represents the mean value of three biological replicates under each treatment combination.

To further explore the major sources of variation in chlorophyll fluorescence parameters, principal component analysis (PCA) was performed across cultivars and drought treatments (**Figure 8**). The first two principal components explained 86.9% of the total variance, with PC1 explaining 63.9% and PC2 explaining 23.0%.

The score plot suggested a separation of cultivars along the PC1 axis. Samples of ‘MD-2’ and ‘Paris’ tended to cluster on the positive side of PC1 and were associated with higher loadings of *NPQ*, *CEF*, *ETR(II)*, and *ETR(I)*, whereas ‘Tainong21’ was positioned toward the negative side of PC1 and was more closely associated with *Y(NO)*.

Along the PC2 axis, samples appeared to be partially ordered according to drought severity. Severe drought treatments were located toward the positive direction of PC2 and were associated with relatively higher *Y(ND)* and *NPQ* values, whereas mild drought and rewatering treatments were generally positioned toward the negative PC2 region and were associated with higher electron transport rates.

The PCA also suggested differences in recovery trajectories among cultivars. Rewatered samples of ‘MD-2’ were located closer to the mild drought cluster, whereas ‘Paris’ showed intermediate shifts and ‘Tainong21’ remained relatively distant from its initial position.

## 4 Discussion

The remarkable drought resilience of pineapple (Ananas comosus), which enables stable productivity under water-limited environments, is generally associated with anatomical and physiological adaptations in its succulent leaves, including high water-storage capacity and diurnal stomatal closure that minimizes transpirational water loss (Borland et al., 2009; Carr, 2012). As a typical CAM species, pineapple also exhibits considerable physiological plasticity under prolonged drought, during which the photosynthetic system may shift toward a low-energy-consumption state known as CAM-idling (Masrahi et al., 2015). Nevertheless, water deficit generally constrains photosynthetic electron transport, leading to reduced PSII activity and activation of photoprotective processes such as non-photochemical quenching (*NPQ*) (Marques et al., 2021).

During drought progression, dynamic reconfiguration of photosynthetic energy partitioning represents an important regulatory process for managing excitation pressure and minimizing photoinhibition. According to the PSII energy partitioning framework proposed by Kramer et al. (2004), absorbed light energy is distributed among photochemical utilization [*Y(II)*], regulated thermal dissipation [*Y(NPQ)*], and non-regulated energy loss [*Y(NO)*]. Among these pathways, increases in *Y(NO)* are often associated with over-reduction of reaction centers and elevated photoinhibition risk. Previous studies have shown that drought-induced stomatal limitation and reduced carbon assimilation in C_3_ crops such as soybean enhance *NPQ* as a major photoprotective pathway, thereby helping to maintain reaction centers in a more oxidized state and protecting PSII from photodamage (Wang et al., 2024). Similar responses have been reported in C4 crops such as maize and sugarcane, where prolonged drought stress induces increases in *NPQ* and alters its kinetic characteristics, often accompanied by changes in *Y(NO)* (Jia et al., 2020; An et al., 2023).

In CAM plants, the characteristic pattern of daytime stomatal closure and nocturnal CO_2_ fixation can exacerbate the imbalance between energy input and carbon assimilation under drought conditions (Osmond, 1978; Borland et al., 2014). Under such circumstances, the induction of relatively high *NPQ* even at moderate irradiance may represent a potential photoprotective response. At the molecular level, drought has been shown to influence thermal dissipation capacity through regulation of *PsbS* abundance, formation of a trans-thylakoid proton gradient (*ΔpH*), and activation of the xanthophyll cycle, thereby contributing to the stability of the photosynthetic apparatus under stress conditions (Pandey et al., 2023; Zong et al., 2023). Manipulation of these photoprotective pathways has even been proposed as a strategy to improve crop water-use efficiency under limited water supply (Turc et al., 2024). The pattern observed in this study—relatively moderate *ETR(II)* accompanied by rapid enhancement of *NPQ*—therefore may reflect the early activation of ΔpH-dependent non-photochemical quenching during the initial stages of drought stress in pineapple. This balance may be particularly relevant in CAM plants, as CAM carbon cycling is associated with higher energetic costs than C3 photosynthesis due to additional processes such as nocturnal CO_2_ fixation and daytime decarboxylation (Lüttge, 2004).

Consistent with observations in many C_3_ and C_4_ species, excess excitation energy during early drought was primarily associated with increased NPQ, as also reflected in the fluorescence responses observed in this study. During moderate drought, *Y(NO)* remained relatively stable, whereas under severe stress *ETR* declined substantially and the capacity for *NPQ* weakened, accompanied by an increase in Y(NO) (Zait et al., 2024). Following rewatering, NPQ and Y(NO) partially recovered toward pre-stress levels, indicating that drought-induced limitations were largely functional rather than structural. Drought-induced inhibition of the photosynthetic apparatus is often reversible, and the extent of recovery after rewatering depends largely on the intensity and duration of stress. When limitations mainly involve metabolic constraints or energy partitioning adjustments, rehydration can restore electron transport and rebalance energy dissipation, resulting in recovery of chlorophyll fluorescence parameters and photochemical efficiency (Pinheiro and Chaves, 2011; Zhang et al., 2025). In the present study, the drought treatment lasted longer than that reported by Zait et al. (2024) (24 d vs. 14 d), yet the photosynthetic apparatus maintained relatively stable regulation until late stages of stress. This suggests that pineapple may endure drought-induced perturbations for an extended period before substantial photosynthetic destabilization occurs.

The persistently high *Y(ND)* observed in this study suggests that PSII may contribute to limitations on the electron supply to PSI during drought progression. Simultaneous increases in *Y(NA)* and *Y(ND)* are consistent with the occurrence of both donor- and acceptor-side limitations in PSI electron transport, which is generally interpreted as a signal of functional restriction rather than irreversible photodamage (Hu et al., 2023). The rapid decline of *Y(NA)* after rewatering further suggests a recovery of downstream electron transport, indicating that no clear evidence of severe irreversible damage was observed. In contrast, studies in maize have shown that after severe drought followed by 10 days of rewatering, *NPQ* failed to recover substantially, while *Y(II)* and *Y(NO)* recovered to less than half of their pre-stress levels (Jia et al., 2020). Compared with these observations, the stronger recovery of energy partitioning observed in pineapple may reflect a relatively high resilience of the photosynthetic apparatus, which may be related to the ecological characteristics of CAM metabolism.

Substantial variation in drought responses has also been reported among genotypes within the same crop species. In maize and wheat, drought-tolerant genotypes tend to maintain more effective regulated energy dissipation and a safer PSI redox balance, whereas sensitive genotypes often exhibit greater susceptibility to photoinhibition under prolonged stress (Bashir et al., 2021; Popova et al., 2023). Different genotypes may also rely on different physiological adjustments to sustain photosynthetic stability, including antioxidant regulation, osmotic adjustment, and repair or turnover of damaged proteins (Li et al., 2021; Hickey et al., 2022). Consistent with these observations, the present study revealed cultivar-dependent differences in photosynthetic energy partitioning in pineapple.

The significant cultivar × drought interaction detected for estimated CEF under high light conditions suggests that alternative electron flow pathways may respond differently among cultivars during drought progression. Such a photoprotective function of alternative electron flow has also been observed in CAM orchids exposed to fluctuating light, where enhanced CEF contributes to maintaining PSI redox balance when downstream electron consumption is restricted (Yang et al., 2022). In CAM plants, daytime stomatal closure and dependence on internally released CO₂ from malate decarboxylation may further increase the imbalance between light-driven electron transport and carbon reduction demand under drought conditions. Under such conditions, cultivar-dependent differences in CEF regulation may contribute to maintaining PSI stability and optimizing energy dissipation under combined drought and high-light stress. However, because CEF in the present study was estimated indirectly from ETR(I) − ETR(II), further validation using more direct approaches, such as electrochromic shift (ECS)-based P515/535 measurements of proton motive force (pmf), ΔpH, and proton conductivity, together with analyses of related protein expression, would be required to clarify the underlying regulatory mechanisms.

Overall, the present study suggests that pineapple cultivars exhibit different photosystem response patterns under drought stress. ‘MD-2’ tended to maintain relatively stable electron transport together with moderate photoprotective dissipation, indicating a comparatively balanced adjustment between photochemical activity and energy dissipation. ‘Paris’ exhibited intermediate responses across most fluorescence parameters, suggesting a relatively conservative adjustment pattern. In contrast, ‘Tainong21’ showed larger fluctuations in several parameters, including elevated *Y(NO)* and variable PSI limitation indices, indicating lower stability of photosystem responses under drought conditions. These observations suggest that drought tolerance in pineapple may be associated with differences in electron transport performance and the relative distribution of energy between PSII and PSI.

## 5 Conclusion

This study shows that drought stress altered PSII photochemistry, thermal energy dissipation, and PSI electron transport, and was associated with changes in photosystem stability in pineapple. Across drought progression and subsequent rewatering, the three cultivars exhibited distinct photosystem response patterns, indicating differences in their ability to balance photochemical energy utilization and photoprotective dissipation. Among them, ‘MD-2’ maintained relatively stable photosystem performance and exhibited the strongest recovery after rewatering, whereas ‘Tainong21’ showed greater susceptibility to drought, with larger fluctuations in fluorescence parameters. These findings provide a physiological basis for evaluating drought responses and drought-tolerance screening in CAM crops.

## Data Availability

The raw data supporting the conclusions of this article will be made available by the authors, without undue reservation.
